# Impact of resilience, learning styles, and instruction quality on medical students’ experiences in microsurgical suturing training: a web-based questionnaire study

**DOI:** 10.1186/s12909-026-09099-6

**Published:** 2026-03-31

**Authors:** Nana Yamakita, Kohei Kanaya, Masahiro Agata, Takumi Maruyama, Yuki Kubota, Akifumi Yokota, Takuya Nakamura, Tetsuyoshi Horiuchi

**Affiliations:** 1 Shinshu University School of Medicine, Matsumoto, Nagano, Japan; 2 Department of Neurosurgery, Shinshu University School of Medicine, 3-1-1 Asahi, Matsumoto, Nagano, 390-8621 Japan

**Keywords:** Resilience, Tachikawa resilience scale, Learning styles, Honey and Mumford questionnaire, Instruction quality, Clinical Clerkship, Microsurgical suturing training

## Abstract

**Background:**

Previous research has noted that resilience, learning styles, and the instruction quality are crucial determinants of medical students’ academic outcomes and motivation during clinical clerkship. However, no study has investigated these factors in their relationship with students’ accomplishment and satisfaction in modules involving substantial hands-on training. This study aimed to examine the associations between resilience, learning styles, sense of instruction quality, and undergraduate medical students’ accomplishment and satisfaction in areas of practical training such as microsurgical suturing.

**Methods:**

A total of 109 undergraduate medical students who participated in microsurgical suturing training during their clinical clerkship between October 2024 and July 2025 were included. Resilience was assessed using the 10-item Tachikawa Resilience scale, and learning styles were evaluated using the Honey and Mumford Learning Styles Questionnaire. Students’ accomplishment and satisfaction with the training and the sense of instruction quality were measured using a questionnaire designed for this study after the training.

**Results:**

Medical students’ resilience was not significantly associated with either accomplishment or satisfaction. By contrast, when trainees gave favorable impressions of the instruction, they perceived the practical training as a more enjoyable and fulfilling experience. Furthermore, a stronger Activist learning preference was found to be significantly associated with higher levels of accomplishment in microsurgical suturing.

**Conclusions:**

These findings indicate that enhancing the sense of instruction quality and incorporating diverse learning approaches may improve medical students’ accomplishment and satisfaction in practical training. Further studies are warranted to identify other factors influencing their accomplishment, satisfaction, and practical performance.

**Supplementary Information:**

The online version contains supplementary material available at 10.1186/s12909-026-09099-6.

## Background

**Fig. 1:**
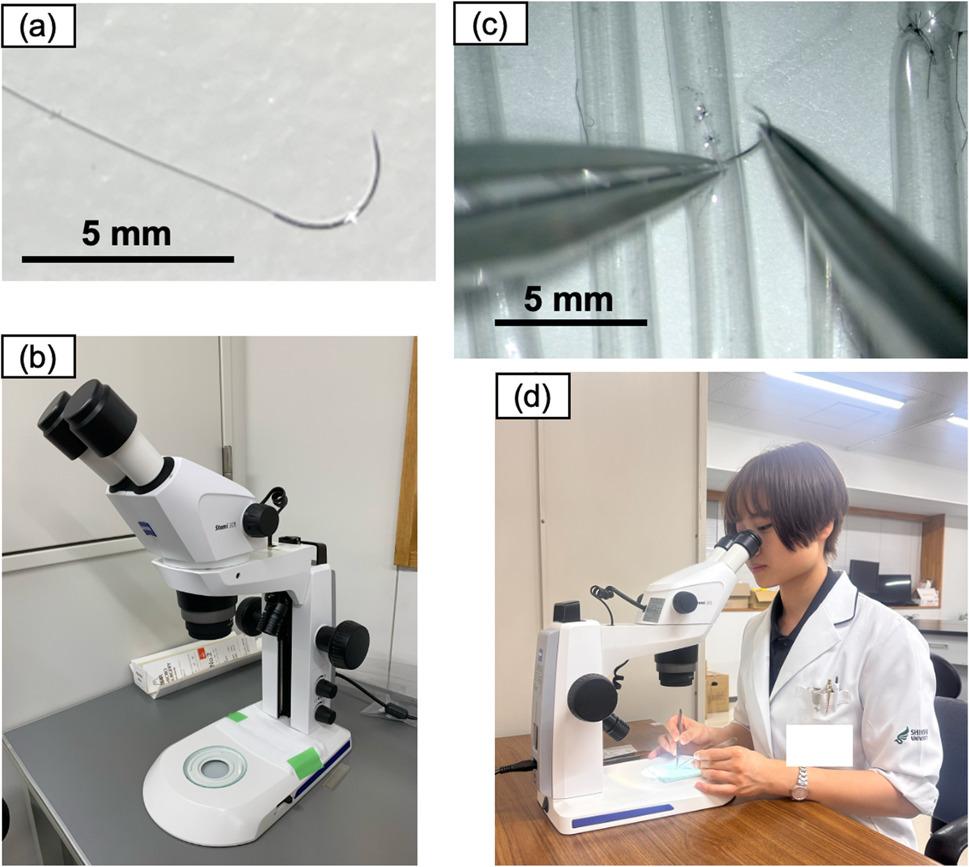
**a** 10-0 nylon and (**b**) Microscope used in this study. **c** Microscopic photographs taken during microsurgical suturing training. **d** Photograph of the first author during microsurgical suturing training

Microsurgical suturing plays an indispensable role in the field of neurosurgical procedures, such as revascularization surgery and nerve reconstruction [1, 2]. We conducted microsurgical suturing training as part of the clinical clerkship in the Department of Neurosurgery at Shinshu University School of Medicine (Fig. 1). Between October 2023 and July 2024, we administered a post-training questionnaire to medical students who had completed the microsurgical suturing training to assess their sense of accomplishment and satisfaction. Based on this preliminary survey, we observed that, due to the technical difficulty and unique characteristics of this training, medical students’ accomplishment and satisfaction varied considerably. We aimed to examine three factors—students’ resilience, learning styles, and the instruction quality—and how these relate to students’ accomplishment and satisfaction with the training.

The first factor is resilience, which has been described in psychology as the capacity to cope effectively with and adapt to adversity or stress, including exposure to such challenges and the attainment of positive outcomes [3, 4]. In recent years, resilience has attracted much attention in the field of medical education [5, 6]. Students with high resilience can manage stress, thrive when facing challenges and continue to develop [7, 8]. Previous studies have reported associations between resilience and academic burnout, life satisfaction, and academic performance among medical students [6, 9–11]. However, to the best of our knowledge, no study has explored its relationship with students’ accomplishment and satisfaction in practical training, such as in microsurgical suturing.

**Fig. 2:**
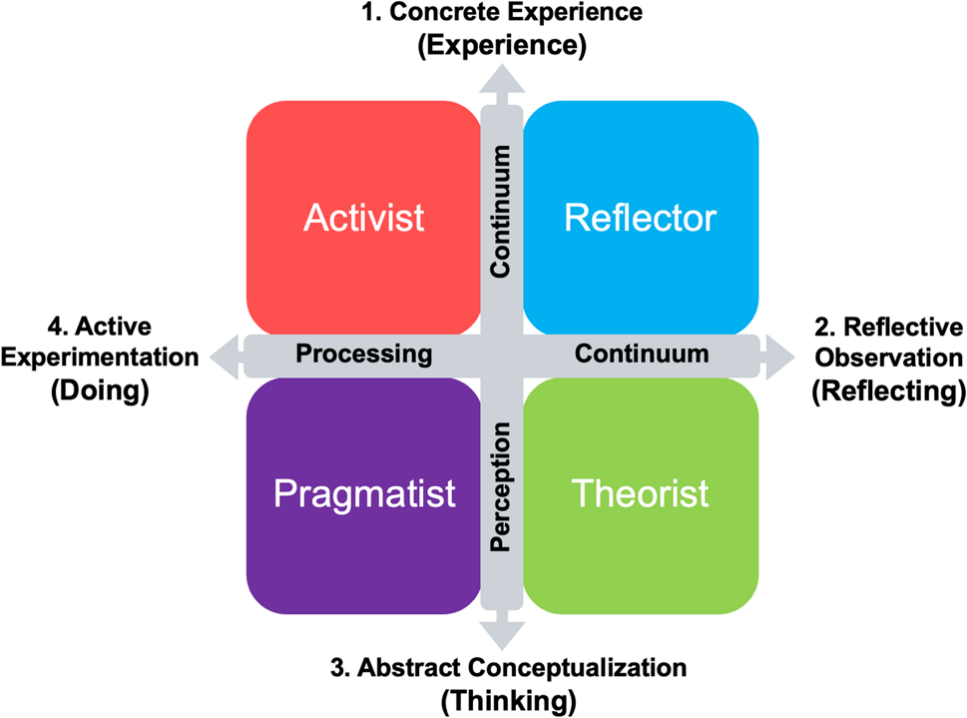
Honey and Mumford Learning Styles Model. This model is based on Kolb’s experiential learning cycle and classifies individual learning styles into four categories according to the two dimensions of the processing continuum and the perception continuum

The second factor is students’ learning preferences, which may influence how they receive and process information [12]. According to Kolb’s experiential learning theory, effective learners must possess four distinct types of abilities: concrete experience (CE), reflective observation (RO), abstract conceptualization (AC), and active experimentation (AE). Based on combinations of these abilities, Kolb conceptualized learning preferences along two dimensions-the horizontal axis (including RO and AE) representing processing, and the vertical axis (including CE and AC) representing perception [13]. Building upon this framework, Honey and Mumford developed the Learning Styles Questionnaire in 1982, which categorizes learners into four types: Activist, Reflector, Theorist, and Pragmatist [14] (Fig. 2). Activists are defined as experimental learners who often dive into tasks without sufficient preparation. They enjoy trying new things and favor learning through trial and error rather than mere observation. Reflectors are thoughtful and introspective learners who analyze situations carefully before making decisions. They learn most effectively when provided with sufficient time and space to process information independently. Theorists are logical and rational learners who seek to understand fundamental principles. They favor organizing information into systematic frameworks and avoid subjective or intuitive approaches. Pragmatists are practical learners who emphasize the practical application of knowledge over abstract theory or principles. They learn most effectively through tasks that require the direct use of newly acquired skills and knowledge [15]. Some studies reported no significant association between learning styles and academic performance among medical students [12, 16]. However, these studies relied on written examination results, without examining learning styles in relation to performance in modules with large practical components.

The third factor is the instruction quality. Previous studies have demonstrated that when trainees form favorable impressions of their educators’ teaching approaches and attitudes, they are more likely to perceive clinical training as an enjoyable and meaningful experience, thereby sustaining their motivation [17–21]. These insights support examining trainees’ perceived instruction quality as an important factor influencing their microsurgical suturing experience.

Therefore, our study aimed to evaluate the impact of resilience, learning styles and the instruction quality on medical students’ experiences in practical training. To this end, we evaluated resilience levels, the learning preferences of medical students, and the instruction quality, and investigated associations with their accomplishment and satisfaction in microsurgical suturing training.

## Methods

### Study design

The study was conducted among undergraduate medical students who participated in microsurgical suturing training during their clinical clerkship in the Department of Neurosurgery of Shinshu University School of Medicine between October 2024 and July 2025. All students had no prior experience with microsurgical suturing training before participating in this study. Before the training, the participants completed an online survey using Google Forms. The questionnaire collected information on various characteristics of the practitioners, including their gender, department of interest (surgical fields, medical fields, or undecided), and resilience. After completing the training, they were asked to voluntarily evaluate their accomplishment and satisfaction and the sense of instruction quality. The total population of eligible students was 109, with 96 providing valid responses (response rate: 88.1%). Students with equally balanced preferences, whose predominant learning style could not be determined (*n* = 8), were excluded. Finally, 88 students were included in the study. This study was approved by the Institutional Ethics Committee on Clinical Investigation of Shinshu University (IRB number 6661), and informed consent was obtained from all the participants.

### Microsurgical suturing training

**Fig. 3:**
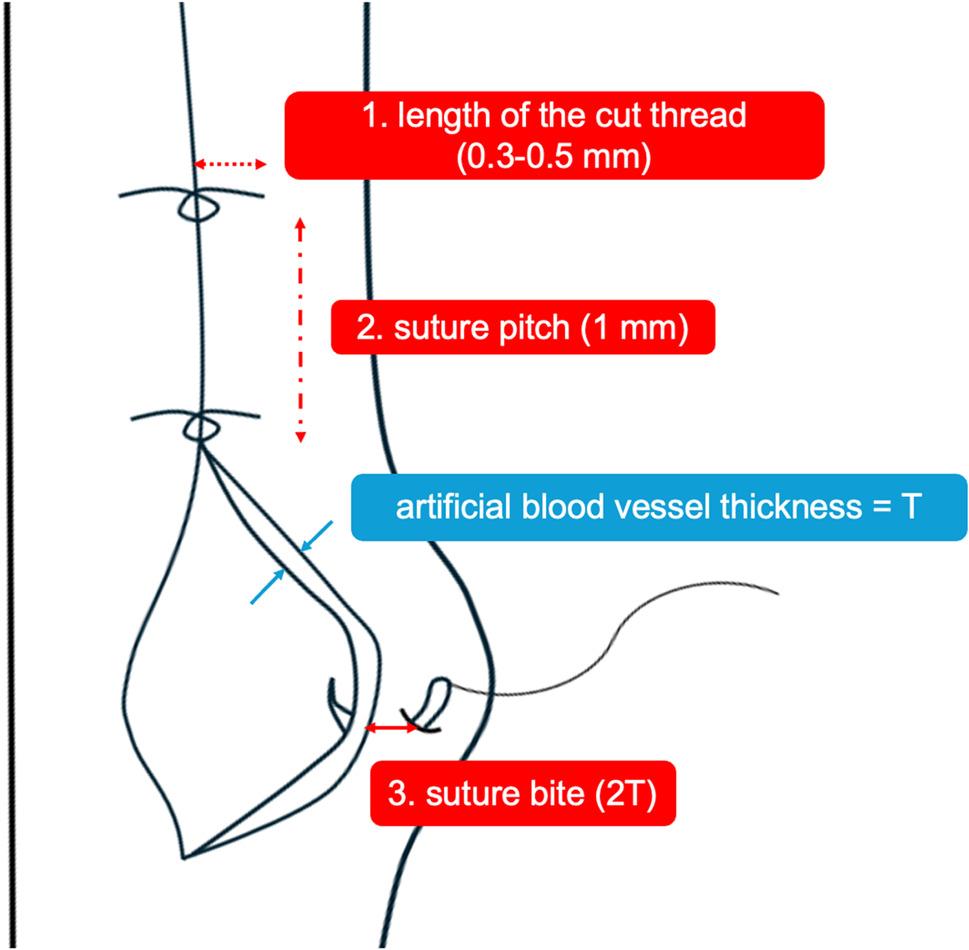
Critical points for high-quality suturing (the most desirable value). “T” means the artificial blood vessel thickness (blue arrow). Point 1 is the length of the cut thread (0.3–0.5 mm) (red dashed double-headed arrow), point 2 is the suture pitch (1 mm) (red dash-dot double-headed arrow), and point 3 is the suture bite (2T) (red double-headed arrow)

This microsurgical suturing training was delivered by two clinical instructors from the Department of Neurosurgery. Students were supervised by one of the two instructors, and the instructor did not change during each student’s training session. Both clinical instructors teach medical students using the same microsurgical suturing training curriculum. At the start of the microsurgical training, participants watched a 3-minute video showing a neurosurgeon performing microsurgical suturing. During the video, the clinical instructor explained key suturing techniques and critical points for high-quality suturing (1. The length of the cut thread, 2. The suture pitch, and 3. The suture bite) (Fig. 3). Subsequently, each student performed microsurgical suturing using forceps, microscissors, and 10 − 0 nylon under the microscope (Zeiss Stemi 305, Carl Zeiss, Germany) for 20 min. Throughout the practice, the clinical instructor provided individualized instruction tailored to each student’s needs. On completion of the 20-minute practice, the clinical instructor provided feedback, marking the conclusion of the training session.

### Resilience

Medical students’ resilience levels were assessed using the 10-item Tachikawa Resilience Scale (TRS) [4]. Each item was rated on a 7-point Likert scale, with students selecting how well each statement described them from 1 (strongly disagree) to 7 (strongly agree), based on their self-assessment. A total score (range, 10–70) was used as an indicator of resilience, and higher total scores reflect higher resilience.

### Learning styles

The Honey and Mumford Learning Styles Questionnaire—translated into Japanese—was used to assess students’ learning styles, namely, Activist, Reflector, Theorist, and Pragmatist [22]. The questionnaire comprised 80 items, and students judged independently whether each item applied to their own experiences or characteristics (responding “yes” or “no”). Each learner’s predominant learning style was defined as the style with the greatest number of “yes” responses, while the strength of each learning preference was quantified by the total number of “yes” responses corresponding to each learning style.

Based on the total scores, the strength of each learning preference was categorized into five levels: very strong, strong, moderate, low, and very low. In particular, the ranges were defined as follows: Activist (13–20: very strong, 11–12: strong, 7–10: moderate, 4–6: low, 0–3: very low), Reflector (18–20: very strong, 15–17: strong, 12–14: moderate, 9–11: low, 0–8: very low), Theorist (16–20: very strong, 14–15: strong, 11–13: moderate, 8–10: low, 0–7: very low), and Pragmatist (17–20: very strong, 15–16: strong, 12–14: moderate, 9–11: low, 0–8: very low). This classification enabled us to analyze not only the predominant learning style of each student but also the strength of their learning preferences across the four domains.

### Medical students’ accomplishment and satisfaction and the sense of instruction quality felt by the trainee

Data were collected using a self-administered questionnaire completed by the participants. Medical students’ sense of accomplishment was assessed using the following eight items: (1) operating the microscope, (2) handling surgical forceps, (3) handling surgical scissors, (4) needle control, (5) passing a needle through a vascular graft, (6) knot-tying, (7) cutting sutures with scissors, and (8) skills improvement compared to before the training. Each item was rated on a 5-point Likert scale, and students rated how well each statement described their accomplishment level from 1 (cannot do it at all) to 5 (able to teach others). The total score (range, 8–40) was calculated, with higher total scores indicating a higher sense of accomplishment. Their satisfaction with the training and the sense of instruction quality were assessed by using separate 5-point Likert scales from 1 (not satisfied at all) to 5 (extremely satisfied), and from 1 (not at all careful or respectful teaching approach) to 5 (extremely respectful teaching).

### Statistical analysis

Descriptive statistics for participant characteristics, including resilience, accomplishment scores, satisfaction scores, and sense of instruction quality felt by trainees, were summarized using means and standard deviations (SD). Because the continuous variables did not meet the assumptions of normality, Spearman correlation coefficients were calculated to examine bivariate associations. To identify independent predictors of sense of accomplishment and satisfaction, multiple linear regression analyses were conducted. The strength of each learning preference (Activist, Reflector, Theorist, and Pragmatist), resilience score, and instruction quality score were included as explanatory variables, with adjustment for gender and department of interest. For each model, unstandardized coefficients (B), standardized coefficients (β), standard errors (SE), and 95% confidence intervals (CI) were reported. All statistical analyses were conducted using Microsoft Excel (Microsoft Corp., Redmond, WA, USA), and a *p* value of < 0.05 was judged to indicate statistical significance.

## Results

A total of 88 medical students were enrolled in our study comprising 53 (60.2%) male and 35 (39.8%) female. Among them, 29 students (33.0%) expressed interest in surgical fields, 38 (43.2%) in medical fields, and the remaining 21 (23.9%) expressed no specific departmental preference. The number of students classified as Activist, Reflector, Theorist, and Pragmatist were 13 (14.8%), 38 (43.2%), 19 (21.6%), and 18 (20.5%), respectively (Table 1). The medical students’ resilience score was 47.9 ± 9.64 (range: 23–70), while the accomplishment score, satisfaction score, and the sense of instruction quality score felt by trainees were 23.8 ± 2.94, 4.09 ± 0.86, and 4.60 ± 0.58, respectively (Table 2).

**Table 1:** Participant Characteristics

Variables		No. (Male/Female)		%
No. of medical students		88 (53/35)		
Department of interest	Surgical Field	29 (20/9)		33.0
	Medical Field	38 (23/15)		43.2
	Undecided	21 (10/11)		23.9
Learning Style	Activist Style	13 (8/5)		14.8
	Reflector Style	38 (22/16)		43.2
	Theorist Style	19 (11/8)		21.6
	Pragmatist Style	18 (12/6)		20.5

**Table 2:** Overall mean of resilience, sense of accomplishment and satisfaction, and instructor quality

	Mean ± SD		
	Total	Male	Female
Resilience (Scale 7-70)	47.9 ± 9.64	47.8 ± 10.4	48.2 ± 8.32
Accomplishment score (Scale 8-40)	23.8 ± 2.94	23.9 ± 2.97	23.5 ± 2.89
Satisfaction score (Scale 1-5)	4.09 ± 0.86	4.04 ± 0.87	4.17 ± 0.84
Sense of instruction quality (Scale 1-5)	4.60 ± 0.58	4.66 ± 0.51	4.51 ± 0.65

*SD* standard deviation

Table 3 shows the strength of participants’ learning preferences, analyzed according to the scoring patterns of the four learning styles described by Honey and Mumford. A total of 30 students (34.1%) showed a “strong” or “very strong” preference for the Activist learning style, while the largest group fell into the “moderate” preference (33 students, 37.5%). Only 6 students demonstrated a “very low” preference (6.8%). In case of the Reflector learning style, 27 students (30.6%) showed a “strong” or “very strong” preference, while 24 (27.3%) and 27 (30.7%) expressed “moderate” and “low” preferences, respectively. A “strong” or “very strong” preference for the Theorist learning style was observed in 23 students (26.1%). However, a larger proportion of students (42 students, 47.7%) showed a “low” or “very low” preference. Furthermore, only 16 students (18.1%) showed a “strong” or “very strong” preference and more than half of the participants (49 students, 55.7%) fell into the “low” or “very low” preference for the Pragmatist learning style.

**Table 3:** The distribution of the strength of participants’ learning preferences

Learning Style	Very strong preference	Strong preference	Moderate preference	Low preference	Very low preference
Activist (%)	16 (18.2%)	14 (15.9%)	33 (37.5%)	19 (21.6%)	6 (6.8%)
Reflector (%)	4 (4.5%)	23 (26.1%)	24 (27.3%)	27 (30.7%)	10 (11.4%)
Theorist (%)	9 (10.2%)	14 (15.9%)	23 (26.1%)	23 (26.1%)	19 (21.6%)
Pragmatist (%)	4 (4.5%)	12 (13.6%)	23 (26.1%)	25 (28.4%)	24 (27.3%)

**Fig. 4:**
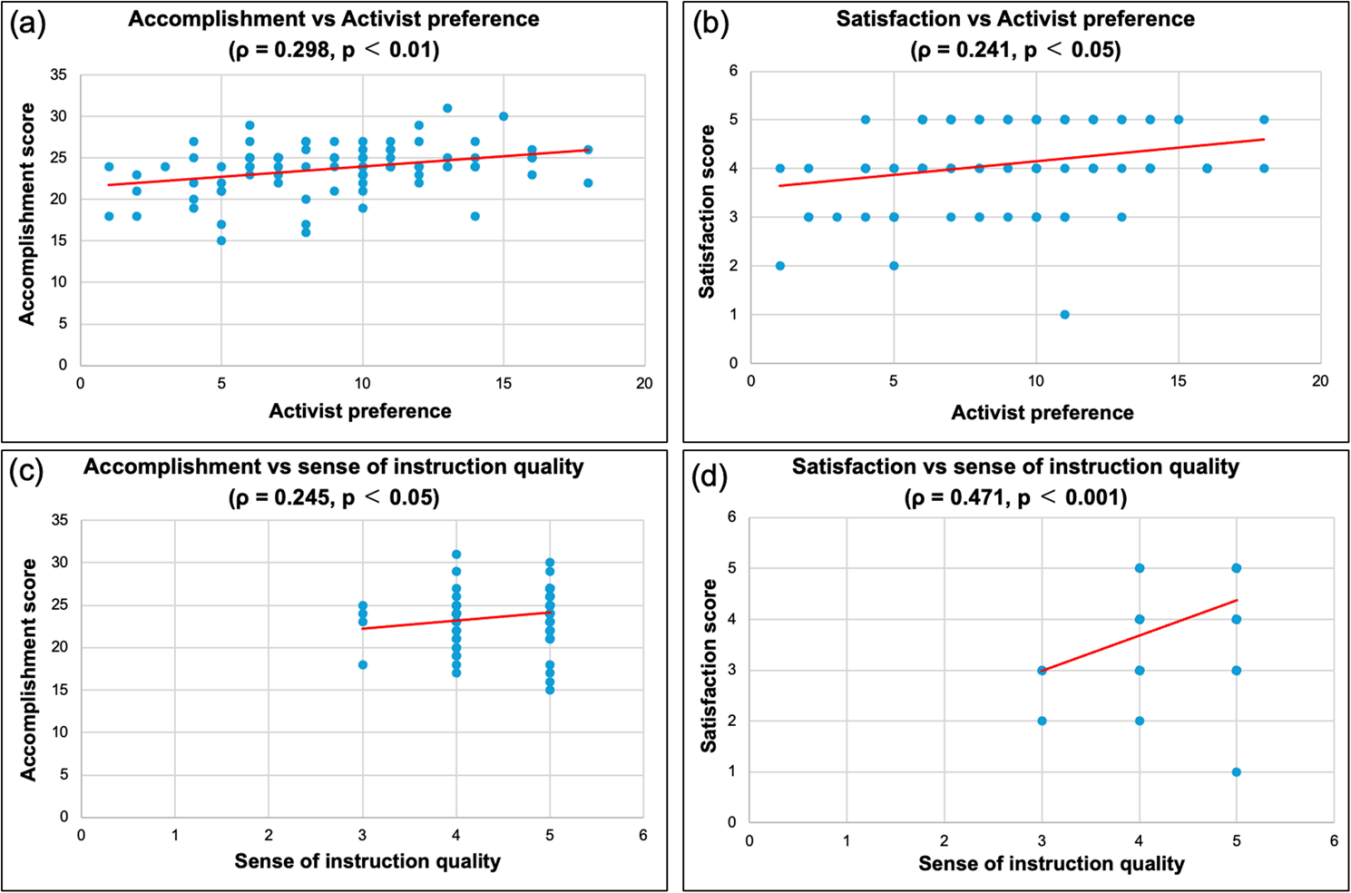
Scatter plots of the relationships between the sense of accomplishment and the strength of Activist learning preference (*ρ* = 0.298, *p* < 0.01) (**a**), the sense of satisfaction and the strength of Activist learning preference (*ρ* = 0.241, *p* < 0.05) (**b**), the sense of accomplishment and the sense of instruction quality (*ρ* = 0.245, p < 0.05) (**c**), the sense of satisfaction and the sense of instruction quality (*ρ* = 0.471, *p* < 0.001) (**d**). Spearman correlation coefficients were calculated. Red lines indicate linear regression fits

Spearman correlation analysis revealed significant positive associations between the strength of Activist learning preference and both sense of accomplishment (ρ = 0.298, *p* = 0.005) and satisfaction (ρ = 0.241, *p* = 0.024) (Fig. 4a, b). The sense of instruction quality also showed significant positive correlations with sense of accomplishment (ρ = 0.245, *p* = 0.022) and satisfaction (ρ = 0.471, *p* < 0.001) (Fig. 4c, d). By contrast, other learning preferences (Reflector, Theorist, and Pragmatist) and resilience were not significantly correlated with either sense of accomplishment or satisfaction (Table 4).

**Table 4:** Spearman correlations for sense of accomplishment and satisfaction predictors

Variable		Accomplishment score		Satisfaction score	
		*ρ*	*p*-Value	*ρ*	*p*-Value
Strength of learning style	Activist	0.298	0.005**	0.241	0.024*
	Reflector	-0.011	0.917	-0.012	0.914
	Theorist	-0.076	0.482	-0.134	0.213
	Pragmatist	0.058	0.594	0.039	0.716
Resilience		0.007	0.952	0.082	0.446
Instruction Quality		0.245	0.022*	0.471	< 0.001***

*p<0.05, **p<0.01, ***p<0.001

Multiple regression analysis was performed to identify independent predictors of sense of accomplishment (Table 5). The overall model was statistically significant (F(6, 81) = 2.25, *p* = 0.046, *R*² = 0.143). Among the predictors, only the Activist learning preference score was independently associated with sense of accomplishment (B = 0.29, 95% CI [0.10–0.47], β = 0.377, *p* = 0.003). Instruction quality, resilience, and other learning preferences (Reflector, Theorist, and Pragmatist) were not significant predictors. Furthermore, multiple regression analysis for predictors of satisfaction revealed that the overall model was also significant (F(6, 81) = 5.03, *p* < 0.001, R² = 0.272) (Table 6). The sense of instruction quality emerged as the strongest independent predictor (B = 0.674, 95% CI [0.38–0.96], β = 0.450, *p* < 0.001). Resilience and learning preferences were not significant predictors. Sensitivity analyses including gender and department of interest as covariates yielded similar results (data not shown).

**Table 5:** Multiple regression analysis for predictors of sense of accomplishment

Variable		B	SE	95% CI	Std. β	*p*-Value
Strength of learning style	Activist	0.29	0.09	[0.10, 0.47]	0.377	0.003**
	Reflector	0.12	0.12	[-0.12, 0.35]	0.126	0.325
	Theorist	-0.04	0.11	[-0.26, 0.19]	-0.045	0.746
	Pragmatist	-0.04	0.11	[-0.25, 0.17]	-0.047	0.696
Resilience		-0.02	0.03	[-0.09, 0.05]	-0.066	0.559
Sense of instruction quality		0.67	0.54	[-0.40, 1.75]	0.131	0.218

*R*² = 0.143 Adjusted *R*² = 0.080, F (6,81) = 2.25, *p* = 0.046*

*B* unstandardized coefficient, *SE* standard error, *CI* confidence interval, *Std. β* standardized coefficient

* *p*<0.05, **p<0.01

**Table 6:** Multiple regression analysis for predictors of sense of satisfaction

Variable		B	SE	95% CI	Std. β	*p*-Value
Strength of learning style	Activist	0.041	0.026	[-0.01, 0.09]	0.186	0.111
	Reflector	0.035	0.032	[-0.03, 0.10]	0.130	0.272
	Theorist	-0.045	0.030	[-0.11, 0.02]	-0.187	0.147
	Pragmatist	0.018	0.029	[-0.04, 0.08]	0.069	0.535
Resilience		-0.007	0.009	[-0.03, 0.01]	-0.083	0.422
Sense of instruction quality		0.674	0.146	[0.38, 0.96]	0.450	*p*<0.001*

*R*² = 0.272, Adjusted *R*² = 0.218, F (6,81) = 5.03, *p* < 0.001*

*B* unstandardized coefficient, *SE* standard error, *CI* confidence interval, *Std. β* standardized coefficient

* *p*<0.001

## Discussion

Microsurgical suturing training is a practical module in which students suture an artificial blood vessel ~ 2 mm in diameter under a microscope, and for most participants, it represents their first such experience. Given its technical difficulty, some students experienced a strong sense of accomplishment and satisfaction, whereas others did not. These observations prompted us to explore which learner-related factors and the sense of instruction quality might underline the variability in students’ experiences during practical training.

### Resilience

Our findings indicated that medical students’ resilience did not influence their sense of accomplishment or satisfaction in microsurgical suturing training. By contrast, previous studies have reported significant associations between resilience and outcomes such as academic performance, academic burnout, life satisfaction, and stress among medical students [6, 9–11]. Possible explanations for this finding include the limited impact of resilience on short-term challenges or difficulties encountered in specific training sessions, and the use of different resilience scales among medical students across studies. A previous study has identified mechanisms through which resilience exerts its effects, demonstrating that university students with higher resilience achieved better academic outcomes through self-management behaviors. This mediating pathway was further reinforced by grit (i.e., perseverance in the face of challenges) and sufficient social support, ultimately contributing to improved academic performance over time [23]. Similarly, Farchi et al. proposed that resilience can be conceptualized as a dynamic process, beginning with the identification of accessible coping resources and the acceptance of situational reality, followed by the constructive reinterpretation of stressors, and culminating in the flexible adjustment of coping strategies to contextual demands [24]. These studies indicated that resilience operates through several sequential stages and exerts its effects over the long term. In this study, microsurgical suturing training was limited to a single 20-minute session, which may have been insufficient for students to demonstrate resilience.

Although resilience is typically conceptualized as the capacity to adapt and perform effectively under stress, the microsurgical suturing training in our study likely represented a different context. Despite the high technical difficulty of the procedure, the training consisted of a single 20-minute session focused on initial skill acquisition. Consequently, it is possible that the training did not impose a substantial level of stress on the students. Resilience may exert a greater influence in situations that require the application of already developed skills under pressure, rather than in brief, structured sessions designed primarily for initial learning. Future studies that examine how resilience functions as students accumulate skills across multiple training sessions may provide clearer insights into the ways in which resilience influences practical performance.

From a cultural perspective, widely used measures such as the 14-item Resilience Scale (RS-14) and the Connor–Davidson Resilience Scale (CD-RISC) may not be optimal for a Japanese population owing to the tendency to suppress positive emotional expression [4, 25, 26]. Accordingly, the TRS was developed as a culturally sensitive measure of resilience in Japanese populations. However, as a relatively new scale, the TRS has been validated primarily in Japanese contexts, and its generalizability to other populations remains to be established [4]. To date, applications of the TRS have been restricted to company workers and patients with psychiatric disorders, and no study has validated its use specifically in student populations [27]. Although the TRS has been used to examine associations between resilience and outcomes such as mental health and life satisfaction, its validity for assessing the relationship between resilience and academic burnout or academic performance remains established [4, 27, 28]. Furthermore, because the TRS differs conceptually from internationally used scales such as the RS-14 and CD-RISC, the comparability of findings across studies may be limited. Further research is needed to determine the appropriate timeframe in which resilience demonstrates its effects on medical students’ accomplishment and satisfaction, and to establish the most appropriate resilience scale for evaluating resilience in Japanese populations.

### Sense of instruction quality felt by the trainee

Our study identified a significant association between the sense of instruction quality felt by the trainee and medical students’ satisfaction. Previous research has shown that when trainees hold favorable impressions of their educators’ attitudes and teaching approaches, they are more likely to perceive clinical training as an enjoyable experience [20]. The current results are consistent with this previous report. Guzman et al., through analysis of trainees’ questionnaire responses, identified one of the most distinctive characteristics of effective clinical instructors is their thorough knowledge of the training content coupled with advanced technical proficiency [21]. In our training program, medical students received instruction from a neurosurgeon with advanced expertise in microsurgical techniques, which may have contributed to their high level of satisfaction. These results indicate the importance of instructors accumulating similar practical teaching experiences and developing more refined instructional skills. Furthermore, Valiee et al. reported that the essential characteristics of effective clinical instructors include fostering students’ independence and confidence, providing active support, maintaining effective communication, and assisting them in identifying and managing their problems [19]. However, in our study, the specific characteristics that remain unclear most strongly influenced students’ satisfaction, as the quality of the clinical instructor was assessed only through separate 5-point Likert scales. Therefore, further research is warranted to elucidate the specific clinical instructor-related factors that influence students’ satisfaction.

### Strength of learning preferences

In this study, no significant differences based on medical students’ predominant learning styles were observed in their accomplishment and satisfaction. However, when an individual’s learning characteristics are regarded as the strength of learning preferences rather than as a distinct style, students with a high Activist learning preference demonstrated a greater sense of accomplishment than those with a low Activist learning preference. Within the Honey and Mumford Questionnaire, classifying an individual’s learning style solely by the category with the highest number of corresponding items did not identify trainee-related factors associated with accomplishment and satisfaction in microsurgical training. In contrast, when the strength of all four learning preferences was evaluated for each trainee, these associations became apparent. The Honey and Mumford Questionnaire assumes that students prefer a unimodal learning style and does not account for the possibility that they may have multimodal learning preferences. On the other hand, previous studies have shown that students acquire multiple learning styles through the process of knowing how to learn and adapting to various approaches [25, 26]. Building on previous research indicating that students may possess multiple learning styles with varying degrees of strength, our study identified the factors influencing students’ sense of accomplishment and satisfaction by evaluating the strength of the four learning preferences within each individual. Our findings suggest that with undergraduate medical students undergoing practical training, evaluating the strength of their learning styles may provide a more efficient analysis than categorizing them into a single learning style.

Furthermore, these results imply that microsurgical suturing training was more consistent with the learning approaches favored by the Activist learning style, which emphasizes direct experience. In this training exercise, slides and textbooks were not utilized; instead, learners engaged in hands-on practice guided by instructional videos and verbal guidance from clinical instructors. While this alignment may help explain why students with stronger Activist preferences reported higher accomplishment, such interpretations remain tentative and should not be taken as evidence of a causal mechanism. This experiential approach might have facilitated more effective learning for students with stronger Activist preferences, potentially contributing to a greater sense of accomplishment. Some studies have reported the value of incorporating various types of modalities into the curriculum to develop students’ academic outcomes [12, 26]. In light of these findings, integrating dynamic and adaptive learning approaches could potentially support students’ sense of accomplishment; however, further research using more objective measures is needed to substantiate this possibility.

### Limitations

There are several limitations to this study. First, only a small number of medical students from a single medical university were enrolled, which limits the generalization of the findings of the research with different training environments. Furthermore, the sampling was based on convenience sampling within a single clerkship cohort, and no a priori power analysis was performed. Post-hoc power analysis indicated that with our sample size of 88 participants and six predictors, we had approximately 80% power to detect medium effect sizes (f² = 0.15) at α = 0.05. However, small effects may have been undetected, particularly for resilience, which showed minimal correlation with outcomes. The non-significant finding for resilience should therefore be interpreted with caution, as it may represent either a true null effect or insufficient power to detect a small effect. More generally, limited statistical power may have contributed to other non-significant associations as well.

Second, all outcomes in this study were based on students’ self-reported evaluations, reflecting perceived rather than actual technical competence. Although self-assessment is suitable for capturing subjective constructs such as “sense of accomplishment,” novice trainees may lack sufficient expertise to accurately evaluate their own performance on specific microsurgical tasks, potentially introducing measurement bias. Future studies should incorporate objective assessments, such as expert evaluation using validated tools (e.g., OSATS), to complement self-reported measures and determine whether the observed associations extend to actual skill acquisition.

Third, the quality of instruction was measured using only a single 5-point Likert item. Given the multifaceted nature of clinical teaching, this single-item subjective measure may be insufficient to fully capture instructional quality. Future research should incorporate more comprehensive and validated instruments.

Fourth, the 20-minute training session was brief and intensely hands-on, which may have inherently favored the Activist learning style while potentially disadvantaging other learning preferences. This format-specific bias should be considered when interpreting the association between Activist preference and positive outcomes. Future studies examining longer training programs with varied pedagogical approaches would help clarify whether these findings generalize beyond brief, experiential learning contexts.

Finally, the association observed between the strength of Activist learning preference and students’ sense of accomplishment may reflect subjective perceptions rather than objective outcomes in microsurgical suturing. Further studies should incorporate objective assessments, longer training durations, and adequately powered study designs to better understand factors influencing medical students’ sense of accomplishment, satisfaction, and practical performance. Several potential confounding variables were not assessed, including prior experience with fine motor skill activities, previous surgical exposure, and baseline dexterity. Given the cross-sectional design of this study, causal relationships cannot be established, and findings should be interpreted as associations rather than causal effects.

## Conclusion

In conclusion, we found that effective clinical instruction and a strong Activist learning preference were related to medical students’ accomplishment and satisfaction among medical students during microsurgical suturing training. These findings underscore the pivotal role of high-quality instruction and indicate the need for dynamic and adaptive learning approaches in microsurgical suturing training. Moreover, given the subjective nature of current evaluation methods, our study highlights the substantial need to develop more objective and standardized assessment strategies for microsurgical suturing training.

## Supplementary Information

Supplementary Material 1.

## Data Availability

The datasets generated and/or analyzed during the current study are available from the corresponding author on reasonable request.

## Abbreviations

CE: Concrete experience
RO: Reflective observation
AC: Abstract conceptualization
AE: Active experimentation
TRS: Tachikawa Resilience scale
SD: Standard Deviation
RS-14: 14-item Resilience Scale
CD-RISC: Connor–Davidson Resilience Scale
