# Supplementary material for: Impact of resilience, learning styles, and instruction quality on medical students’ experiences in microsurgical suturing training: a web-based questionnaire study
Source: BMC Med Educ. 2026 Mar 31;26:745. doi: 10.1186/s12909-026-09099-6 (PMC13162420; doi:10.1186/s12909-026-09099-6)
Supplement: Supplementary file 1 — Supplementary Material 1. [file 12909_2026_9099_MOESM1_ESM.docx]

# Questionnaire for Microsurgical Suturing Training

Please cooperate with this self-assessment questionnaire on microsurgical suturing training.

1. **Microscope operation**

Select only one.

1. Cannot do it at all
2. Cannot do it well
3. Can do it
4. Can do it well
5. Can teach others
6. **Forceps operation**

Select only one.

1. Cannot do it at all
2. Cannot do it well
3. Can do it
4. Can do it well
5. Can teach others
6. **Scissors operation**

Select only one.

1. Cannot do it at all
2. Cannot do it well
3. Can do it
4. Can do it well
5. Can teach others
6. **Needle control**

Select only one.

1. Cannot do it at all
2. Cannot do it well
3. Can do it
4. Can do it well
5. Can teach others
6. **Passing needle through artificial vascular graft**

Select only one.

1. Cannot do it at all
2. Cannot do it well
3. Can do it
4. Can do it well
5. Can teach others
6. **Tying the thread**

Select only one.

1. Cannot do it at all
2. Cannot do it well
3. Can do it
4. Can do it well
5. Can teach others
6. **Cutting the thread with scissors**

Select only one.

1. Cannot do it at all
2. Cannot do it well
3. Can do it
4. Can do it well
5. Can teach others
6. **Microsurgical suturing skill after the training**

Select only one.

1. Did not improve at all
2. Did not improve much
3. Improved slightly
4. Improved considerably
5. Improved greatly
6. **Satisfaction with the microsurgical training**

Select only one.

1. Not satisfied at all
2. Not very satisfied
3. Somewhat satisfied
4. Satisfied
5. Very satisfied
6. **How was the teaching method?**

Select only one.

1. Not careful teaching at all
2. Not careful teaching
3. Average
4. Taught carefully
5. Taught very carefully
